# Annual Meeting of the Belgian Society of Radiology (BSR) November 19, 2016

**DOI:** 10.5334/jbr-btr.1241

**Published:** 2017-01-05

**Authors:** Maryam Shahabpour, Walter De Wever

**Affiliations:** 1 University Hospital Brussels, BE; 2 University Hospitals Leuven, BE

**Keywords:** belgian society of radiology, musculoskeletal imaging, thorax imaging, shoulder instability

## Abstract

Editorial on the Annual Meeting of the Belgian Society of Radiology (BSR) 19 November 2016.


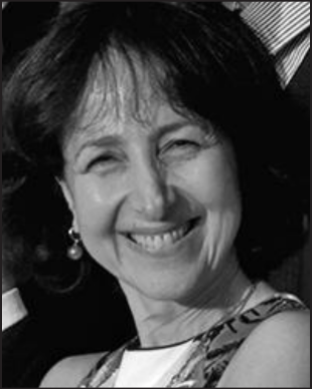
    Maryam Shahabpour


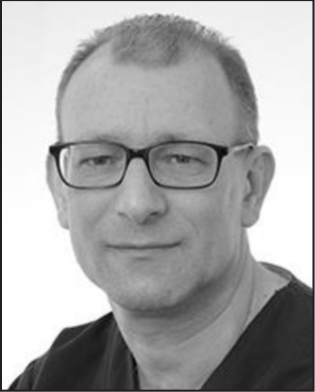
    Walter De Wever

The BSR Meeting is focused this year on two very important disciplines in daily practice: **Musculoskeletal and Chest Imaging**.

**1.** The **Musculoskeletal Radiology Sessions** are organized by **Maryam Shahabpour** (Chair of the MSK Radiology Section of BSR) and **Laurens Topff** (Chair of the Young Radiologist Section of BSR).

**1.1** The first 90-minute session on **Musculoskeletal Imaging** focuses on **Miscalls and Mimics** as proposed by the Young Radiologists Section and is chaired by **Rita Lopes do Rosário** (from the French Catholic University of Louvain UCL) and **Veerle De Grove** (from the Dutch Vrije Universiteit Brussel VUB).


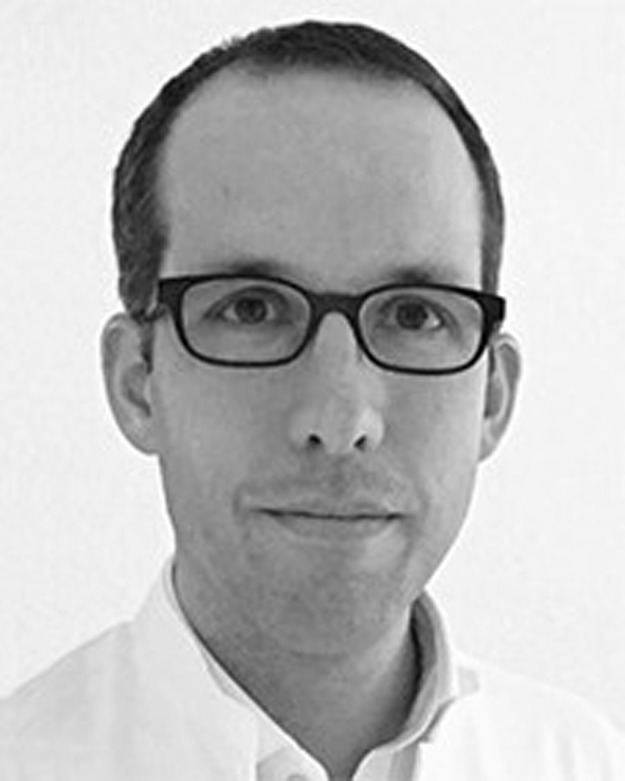
    Reto Sutter

The first invited speaker is Doctor **Reto Sutter**, radiologist and PD, Deputy Head of the Radiology Department at the well-known Orthopedic University Hospital Balgrist in Zurich/Switzerland. Dr. Sutter graduated from medical school at the University of Zurich and trained in radiology and pathology in the University Hospital Zurich and the Cantonal Hospital in Winterthur as well as in the Royal London Hospital/Queen Mary University in London, United Kingdom. During his training, his research focus was on MRI of the abdomen and the cardiovascular system. He further investigated neural stem cells and their role in the development of medulloblastoma during his stay in the UK.

His main research interests are imaging of the joints, imaging of metal hardware and reduction of metal artifacts on CT and MR imaging. Dr Sutter published several widely read studies on femoroacetabular impingement and further published several studies on metal artifact reduction at MRI, e.g. in patients with total hip and total knee arthroplasty, both from a technical and a clinical view point. At Balgrist Hospital, more than 90% of his work is focused on musculoskeletal radiology. As such, he performs many CT and MR arthrography examinations, e.g. in shoulder instability, but also examines a lot of high-level athletes from different sports activities. He has published 48 peer-reviewed papers (on PubMed) since 2007 (17 as the first author). Dr Sutter received several awards for his work, including the 2012 Jubilee Award of the Swiss Society of Radiology. In 2014, he received his habilitation (formal initiation as senior lecturer in the medical faculty at the University of Zurich). He is a member of the assistant editorial board for the Journal *Investigative Radiology* and is acting as reviewer for a number of prestigious journals, including *Skeletal Radiology* and *Radiology*. He is a member of the International Skeletal Society and holds the Diploma of the European Society of Musculoskeletal Radiology (ESSR), where he is also a member of the ESSR Sports Imaging Subcommittee (chaired by Maryam Shahabpour since 2013).

Reto Sutter approaches “**Sports Injuries**” through the radiological misinterpretations that the radiologists should learn to recognize and to avoid. Athletes can present a variety of common and specific injuries. Some of the sports injuries are initially misdiagnosed, leading to possible mid- and long-term secondary complications that may delay return to sports or prevent the athlete from competing at an elite level. Furthermore, normal physiological and mechanical phenomenons in athletes could be interpreted as abnormal imaging findings, especially in young athletes. As an example, an athlete with acute tear of the anterior cruciate ligament of the knee could present associated injuries to the joint capsule and the posterolateral or posteromedial stabilizing structures. If those lesions are missed and left untreated, the athlete could develop a posterolateral or posteromedial instability of the knee joint. Additionally, there are more typical injury patterns and locations diagnosed in specific sports, such as subtalar fracture of the lateral process of the talus, the so-called snowboarder’s ankle,that could be missed on plain radiographs and sometimes on MR imaging. On the other hand, there are seemingly abnormal findings in athletes detected on radiographs, CT or MR imaging that are commonly encountered but completely asymptomatic. Professor Sutter concludes that a good communication between the referring sports physician and the radiologist is paramount to provide a fast and correct diagnosis, seeing the patient history and clinical examination helps to avoid misinterpretation of the imaging findings.


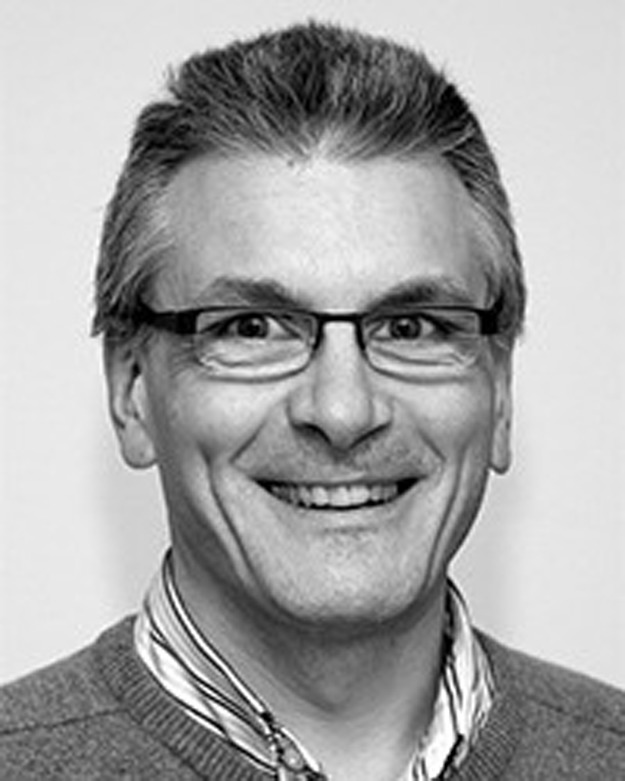
    Filip Vanhoenacker

The second speaker of the first session is Doctor **Filip Vanhoenacker**, Belgian radiologist who studied at the Catholic University of Leuven. He is staff radiologist at the General Hospital of St-Maarten, Mechelen-Duffel since 1991 and consultant radiologist at the University of Antwerp since 1995, as well as guest lecturer since 2007 and guest professor since 2013. He is also guest professor at the University of Ghent since 2011. In 2003, he obtained a PhD based on “congenital abnormalities of the skeleton”, one of his numerous fields of interest. He has published 296 peer-reviewed papers referenced on PubMed and is the author or co-author of 61 book chapters and monographs. He is the editor and co-editor of “Imaging of Soft Tissue Tumors” by AM De Schepper, PM Parizel, L De Beuckeleer, F Vanhoenacker 2001, 2006, and a forthcoming 2017 edition (Springer-Verlag) and “Imaging of Orthopedic Sports Injuries” by FM Vanhoenacker, M Maas, JL Gielen, 2007 (Springer-Verlag). He is a referee for European Radiology, JBR-BTR/JBSR, European Journal of Radiology, Skeletal Radiology, Singapore Medical Journal, British Journal of Sports Medicine, Eurorad, and Insights into Imaging.

The impact of his research topics on the present and future practice of radiology is mainly educational, launching the careers of young radiologists and residents.

Filip Vanhoenacker presents the “**Common Pitfalls in MR Imaging of the Knee Joint**”, an overview of common interpretation errors and pitfalls encountered by young residents or less-experienced radiologists. MRI of the knee joint is one of the most commonly requested examinations and belongs to the core clinical practice in most MRI clinical units (together with spinal and brain MRI). Therefore, these examinations are often reported by general radiologists in most private institutions and are an important part of routine education of radiology residents. Professor Vanhoenacker emphasizes pitfalls due to *insufficient knowledge of anatomic variants* from osseous origin (as bipartite patella or dorsal defect of the patella) that should not be confused with osteochondritis dissecans, Brodie abscess or bone tumors. Cortical avulsive irregularity, another benign bone defect located at the posteromedial femoral condyle should not be misinterpreted as an aggressive neoplastic lesion. He recommends the following to avoid common mistakes:

- Consider *age and clinical findings* as well as previous medical history and symptoms before interpreting the images.
- Use *standardized questionnaires* to indicate the precise location of the pain, duration of the complaints, history of trauma, aggravating activities, underlying diseases and previous surgery.
- Look at *previous imaging studies* and *other imaging modalities*, such as plain films or CT scan (namely for identification of calcifications) or ultrasound, since the comparison with these examinations can be extremely helpful for the correct diagnosis.
- Analyze all imaging planes systematically for all structures and compare the T1 and T2 weighted pulse sequences.
- Look for other clinically atypical abnormalities (as pigmented villonodular synovitis, gout, other crystal deposition diseases, bone marrow abnormalities, ganglion cyst of the anterior cruciate ligament) apart from common intra- and peri-articular pathologies (like menisci, anterior cruciate ligament, cartilage).
- Keep *technical artifacts* in mind as a cause of interpretation errors.
- Overcome the phenomenon of satisfaction of search, where detection of one abnormality may reduce the detectability of another abnormality.

Filip Vanhoenacker recommends a systematic approach in the analysis of all intra- and extra-articular structures of the knee joint.


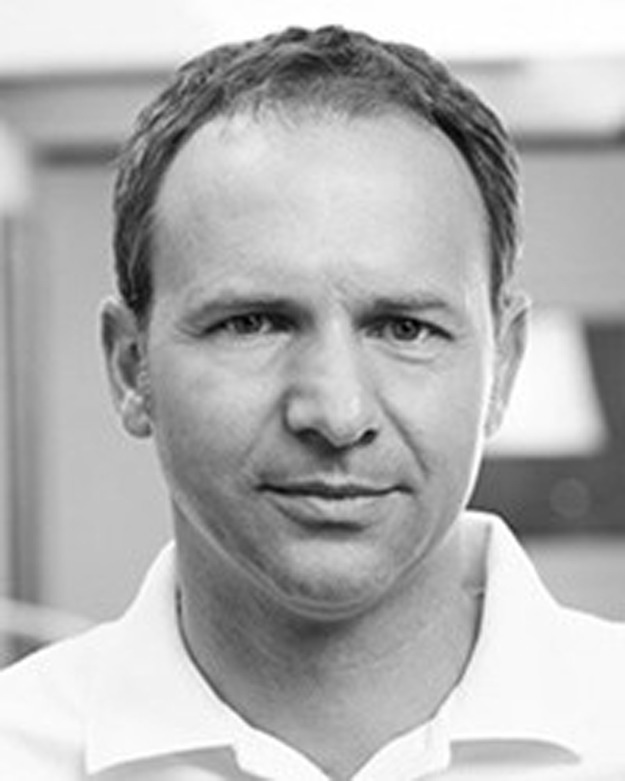
    Christian Glaser

The third speaker of this session is Doctor **Christian Glaser**, radiologist at LM University Munich, who from 2002 to 2009 served as section chief at the Department of Clinical Radiology of LMU Grosshadern. From 2009 to 2011 he was appointed visiting associate professor at the Department of Radiology of New York University NYUMC. Since then he has worked at the Radiology Center Munich. Being trained as a general radiologist, Dr. Glaser has a strong clinical and scientific interest in MSK imaging with special emphasis on quantitative parametric imaging of cartilage in osteoarthritis, imaging of disease activity in rheumatoid disorders and 3D isotropic imaging in MSK. After his thesis on cartilage microarchitecture (SEM) in 1998, he completed his PhD on “quantitative cartilage MRI” in 2007 and has been appointed as APL-Professor of Radiology at LMU Munich in May 2014. Christian Glaser is a passionate teacher who actively contributes to national and international teaching courses and teaching journals. Beyond research and clinical imaging, Dr Glaser is also interested in workflow analysis and optimization in radiology. He obtained the International Skeletal Society President’s medal at ISS 2012 and a monetary grant as special recognition of his outstanding scientific achievements on an international level, under 45 years of age.

When asked what teaching means to him, Christian replies that first of all, teaching is fun and very instructive. It is also a way to give back some of what he had the luck to learn from the teachers he came across. For Christian Glaser, a key feature of radiology as a medical specialty is that the radiologist and his team work at the interface between a more and more subspecialized clinical environment, our patients and a highly sophisticated imaging technique. This comes with the responsibility to continuously and carefully evolve and adapt our position between the poles of a diagnostic service provider, of being active in therapy, and of taking responsibility in patient management. For Professor Glaser it is important to bring to life the interfacing characteristics of our profession, radiology, in day-to-day practice. This implies to combine and integrate at least three aspects: a sound image quality, scrupulous reading and the clinical context.

Christian Glaser develops some of the important errors in imaging of the ankle. His lecture is focused on “**Tarsal Coalitions, a Practical Approach to a Not-so-rare Entity**”.

He explains that the term *coalition* refers to a connection between two normally separate bones, presumably due to a disturbance in mesenchymal segmentation. A coalition may be bony, fibrous, cartilaginous or a mixture of these tissues and may be partial or complete, i.e. it may affect a complete joint (facet) surface area or a fraction of it.

According to Professor Glaser and the scientific literature, imaging should be based on radiographs followed by MRI and – if relevant for therapy – by CT. The role of imaging is to corroborate the suspicion of a coalition, to bring up a coalition as a potential differential diagnosis to explain unclear or prolonged pain, to determine the nature, extent and location of one or more coalitions as well as potential accompanying changes (degenerative or stress reactions, e.g. bone marrow edema pattern), in order to help guide therapy.

The technical evolution of MRI enables this modality to depict and accurately describe any coalition and its morphologic features, reducing the need for CT in the work-up of coalitions. Both CT and MRI have greatly facilitated the diagnosis of tarsal coalitions compared to radiography; this is also reflected by the higher incidence of coalitions in cross-sectional imaging studies. MRI shows *bone marrow edema pattern* around a non-osseous coalition or around adjacent joints. And of course, MRI may demonstrate associated soft tissue changes such as tendon pathologies in foot deformity or sinus tarsi syndrome. In terms of MR image analysis it is helpful to systematically look for the presence or absence/disturbance of the *typical sequence of linear patterns reflecting a joint*: trabecular bone, subchondral bone plate, articular cartilage and joint space. This approach may help to avoid overlooking subtle coalitions. CT is useful in specific situations, such as pre-operatively, to decide whether a resection or arthrodesis is feasible, to specifically plan a procedure, to assess fine bony details of a coalition, or to assess secondary subtle degenerative change. It may also be especially helpful to detect small bony bridges outside/at the very periphery of the main joints of the foot, so-called extra-articular coalitions.

The last speakers of the first MSK session are from the Young Radiologist Section (YRS), a subdivision of the Belgian Society of Radiology (BSR) dedicated to residents and also recently (less than five years) graduated radiologists. Doctor **Solenne Lanotte** (current YRS French speaking Chair from UCL) and Doctor **Cédric Bohyn** (YRS Secretary from KUL) have prepared an original case-based presentation: “**The Hips Don’t Lie: A Case-based Quiz**”.


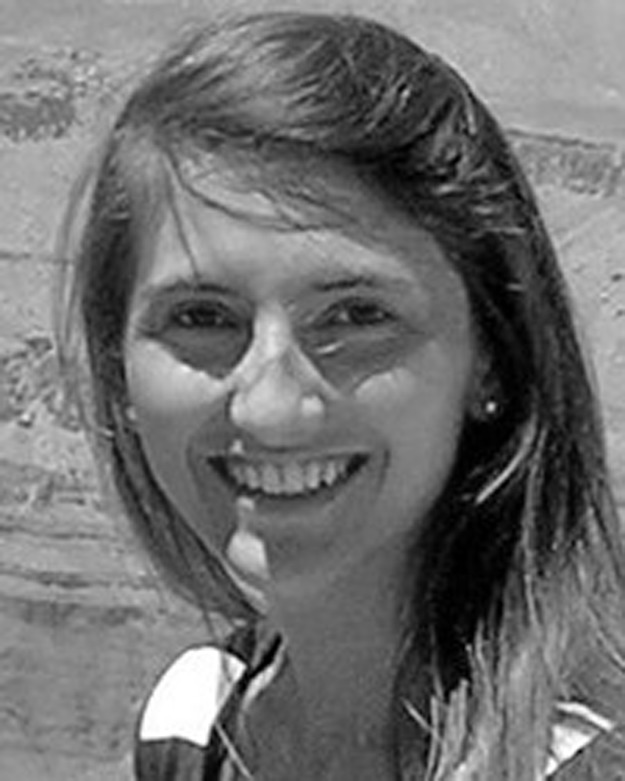
    Solenne Lanotte


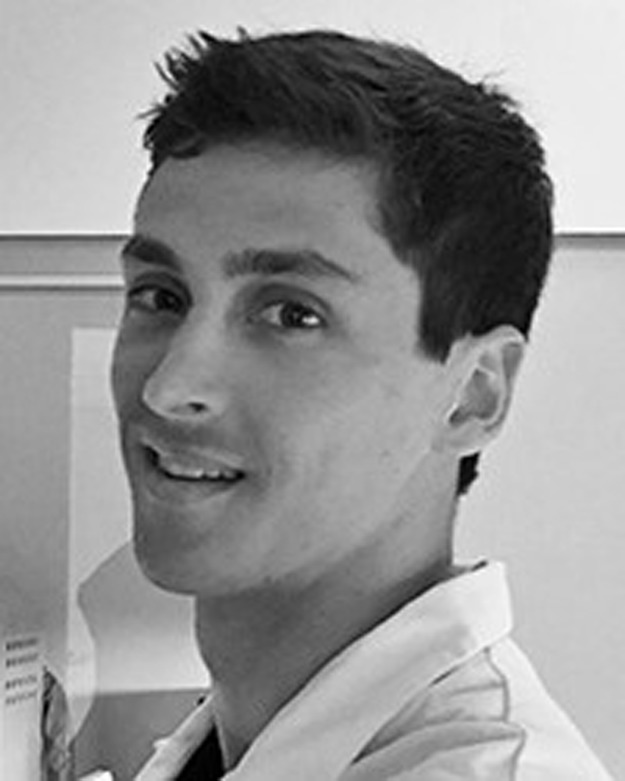
    Cédric Bohyn

To complete the musculoskeletal contributions of this special November issue of the Journal of the BSR, **Anagha Parkar**, radiologist from Bergen, Norway (presenting a lecture at the chest session) was asked to write a paper on her field of interest in musculoskeletal imaging, in particular the role of CT imaging after reconstruction of the anterior cruciate ligament, part of her future PhD thesis.

**1.2** The purpose of the second session on **Musculoskeletal Imaging** is to try to answer to the questions arised in managing patients with **Shoulder Instability: How to Do or Not to Do? and How to Help the Surgeon?** It is organized by the Musculoskeletal Section, chaired by Maryam Shahabpour from the Vrije Universiteit Brussel (VUB). She has invited experts in shoulder imaging from Switzerland (Reto Sutter and Patrick Omoumi) and Bruno Vande Berg from UCL as well as Nicole Pouliart, a skilled shoulder surgeon from VUB Brussels.


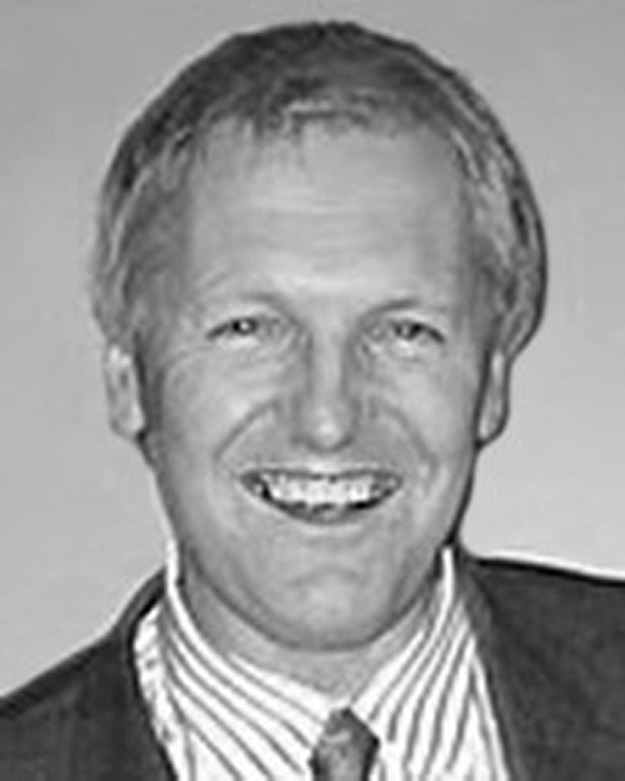
    Bruno Vande Berg

The first speaker, Doctor **Bruno Vande Berg**, is staff radiologist at the Department of Medical Imaging of the University Hospital UCL St Luc since 1994. He obtained a PhD in 1997 on “sequential quantitative analysis and mapping of the bone marrow with MR”, and started in 1998 as a clinical teacher at the Université Catholique de Louvain; in 2004 he became clinical professor responsible for the osteoarticular radiology unit and Chief of the Radiology Department of the Cliniques Universitaires St Luc – UCL. Since 2014, he has been Associated Chief of the Radiology Department, responsible for education and the osteoarticular radiology unit, Chairman of the interuniversity DES (Diploma of specialized studies). He is also Chair of the French Accreditation Committee in Radiodiagnosis.

He obtained research funds from FNRS (Fonds de la Recherche Scientifique) and was scientific collaborator and promotor of the “Télévie” operation from 1990 to 1997 working on “Integration of MRI for evaluation and therapeutic monitoring of leukemia and myeloma”. He obtained the President’s Medal of the International Skeletal Society in 2004 (as did Christian Glaser in 2012). He published about 158 peer-reviewed papers referenced on PubMed, many of them with his mentors Jacques Malghem (212 on PubMed) and Baudouin Maldague (164). Bruno Vande Berg also wrote more than 75 book chapters.

Professor Bruno Vande Berg presents “**Radiographic Analysis of Osseous Injuries after Dislocation of the Shoulder Joint**”. He states that radiography remains pivotal to the workup of instability lesions of the shoulder, both in the acute as well as the chronic settings. The goal of radiography is to detect osseous abnormalities and locate them in order to determine the direction of instability. In anteroinferior instability, Hill-Sachs lesions are often visible at radiography and should not be confused with various differential diagnoses, which are usually more laterally located (as synovial inclusion cysts and marginal inflammatory erosions). Bankart lesions are more difficult to detect at conventional radiography, but there are less false positives than for Hill-Sachs lesions. The Garth view represents an excellent radiographic view to detect anteroinferior instability impaction fractures at both the humeral and glenoid sides. According to Bruno Vande Berg and many MSK experts, conventional radiography is the first step of the diagnostic workup since it allows the detection of osseous abnormalities following dislocations. However, advanced cross-sectional imaging techniques (MR, CT and US) will be required to accurately quantify these bone abnormalities and to detect associated lesions of soft tissue stabilizers of the shoulder (chondrolabral, capsuloligamentous structures) as well as rotator cuff lesions.


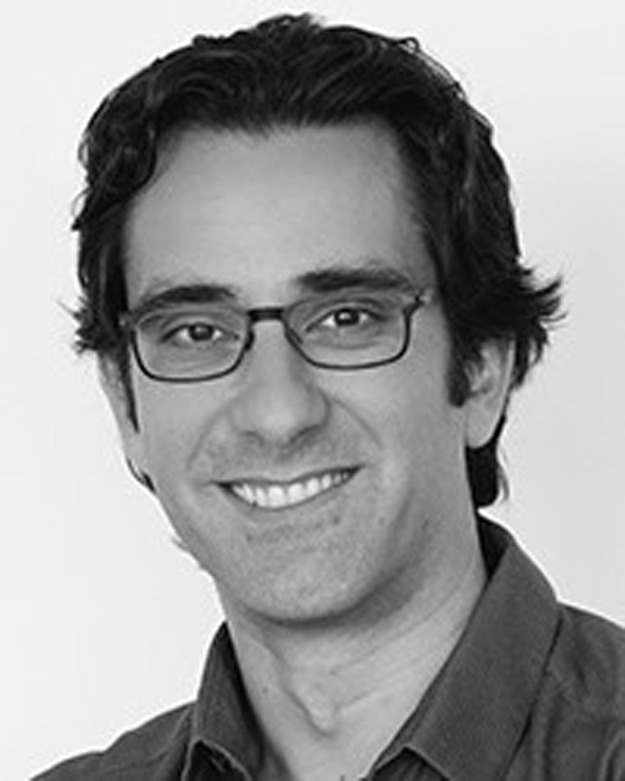
    Patrick Omoumi

The second speaker of the shoulder session is Doctor **Patrick Omoumi**, a radiologist from Persian origin. He graduated from La Pitié-Salpêtrière School of Medicine, Paris, France (2003), and specialized in radiology at the University Hospital of Tours, France, until 2008. After two fellowships in St Luc University Hospital (UCL) in 2008 and the University of California, San Diego (UCSD), in 2009, he joined the Musculoskeletal Radiology team at St Luc University Hospital as a staff radiologist in 2010. Since 2014 he has been the Head of the Musculoskeletal Imaging Unit and Lecturer at the Department of Diagnostic and Interventional Radiology of Lausanne University Hospital, Switzerland. Shortly after his arrival in Lausanne, he started a collaboration at the Swiss Biomotion Lab. He obtained his PhD in UCL in 2015 on “cartilage in some areas of the knee are thickened in osteoarthritis”. He got several international grants and awards. He is reviewer for eight high-impact scientific journals and associate editor of BMC Musculoskeletal Disorders. He was guest editor of two issues of *Seminars in Musculoskeletal Radiology*. He published over 65 papers in peer-reviewed journals, over 100 abstracts/presentations at international conferences, including several awards and 22 book chapters and monographs. He has presented over 50 invited lectures at national and international conferences and has organized the annual symposium on MSK radiology in Lausanne University since 2014. He co-organized the bi-annual joint meeting of the University Hospitals of Brussels (UCL) and Lille on Musculoskeletal Imaging from 2010 to 2014. He is faculty member of some of the MSK Erasmus Courses on Magnetic Resonance Imaging and the European School of Radiology courses.

Professor Omoumi presents “**MR Arthrography in Glenohumeral Instability: It May Not Be as Complicated as It Seems**”. Glenohumeral joint instability is usually an intimidating topic for most radiologists, due to both the complexity of related anatomical and biomechanical considerations, as well as the increasing number of classifications and acronyms reported in the literature. Patrick Omoumi aims to demystify glenohumeral instability by focusing on the relevant anatomy and pathophysiology and reviewing the important imaging findings, and he explains how to describe them for the clinician in a relevant and simple way. The role of the radiologist in assessing glenohumeral instability lesions is to properly describe the stabilizing structures involved (bone, soft tissue stabilizers and their periosteal insertion), to localize them and to attempt to characterize them as acute or chronic. It is less important to find the acronym associated to a lesion. Impaction fractures on the glenoid and humeral sides are important to specify, locate and quantify. Any associated cartilaginous or rotator cuff tendon lesion should be reported to the clinician.

As do most authors, Patrick Omoumi agrees that proper assessment of glenohumeral labroligamentous structures requires the intra-articular injection of contrast material, either with MR- or CT-arthrography, in order to distend the joint cavity. MR and CT arthrography have shown similar (or slightly superior performance for CT arthrography, depending on the studies) for the detection of labral and/or ligamentous lesions, as well as for associated lesions, including cartilage and rotator cuff tendon lesions. For the assessment of bony structures (for which CT remains superior, despite recent progress made at MRI thanks to 3D sequences). However, whenever possible, MR arthrography should be preferred due to the exposure of patients to ionizing radiation with CT, and the proximity of radiosensitive organs such as the thyroid. It has been suggested that additional acquisitions with the shoulder placed in different positions, such as the ABER position, may be useful to improve the detection of antero-inferior labro-ligamentous lesions. For Dr Omoumi and others, the systematic use of the additional positions in the general population may not be required in practice due the low diagnostic yield, the additional examination time and patient discomfort.

The third speaker on shoulder imaging is Doctor **Reto Sutter** (from Balgrist University Hospital, Zurich) who was introduced in the beginning of the editorial.

Professor Sutter shares his experience on “**The Role of CT Arthrography in Shoulder Instability**”. Plain CT and CT arthrography are useful tools in evaluating both the osseous structures and the soft tissues in patients with shoulder instability.

In patients with classic anteroinferior shoulder instability, CT can easily diagnose and quantify the common Hill-Sachs defect and glenoid rim fractures. It is the most accurate modality to assess the glenoid bone. Whereas the amount of osseous defects and glenoid bone loss at the anterior part of the glenoid may be underestimated at MRI in patients with anteroinferior shoulder instability, CT allows a precise visualization of this part of the glenoid, both in acute glenoid fracture and in chronic instability. In cases with only minor glenoid bone loss a labral repair and capsular surgery may be performed, while in cases with substantial glenoid bone loss, usually an osseous corrective surgery is preferred. Further, Reto Sutter reports that CT is a simple modality for performing anatomical measurements in the shoulder, such as glenoid version, or for the assessment and quantification of the amount of osseous deficiencies of the posterior glenoid in patients with suspected posterior shoulder instability. CT is also often used to assess atrophy and fatty infiltration of the rotator cuff muscle. Finally, CT is also beneficial for assessing patients in the postoperative situation, e.g. after a Latarjet procedure, where the distal part of the coracoid process is transferred to the anterior portion of the glenoid in order to prevent re-dislocation of the shoulder joint. CT allows for accurate assessment of the position of the osseous block and detection of a possible non-union. In patients with a suspected dislocation of anchors after rotator cuff repair, CT allows the identification and localization of the anchors and surgical wires.

The injection for CT arthrography can be performed under fluoroscopy guidance, under sonography, or even directly on the CT examination table with a low-dose CT protocol for the injection itself, followed by the standard diagnostic CT. With its inherent high spatial resolution, CT arthrography allows a precise evaluation of labral and chondral defects and is useful for the assessment of the biceps anchor and capsule-labrum complex.

The speakers of the last presentation on shoulder instability are Drs Nicole Pouliart and Maryam Shahabpour.


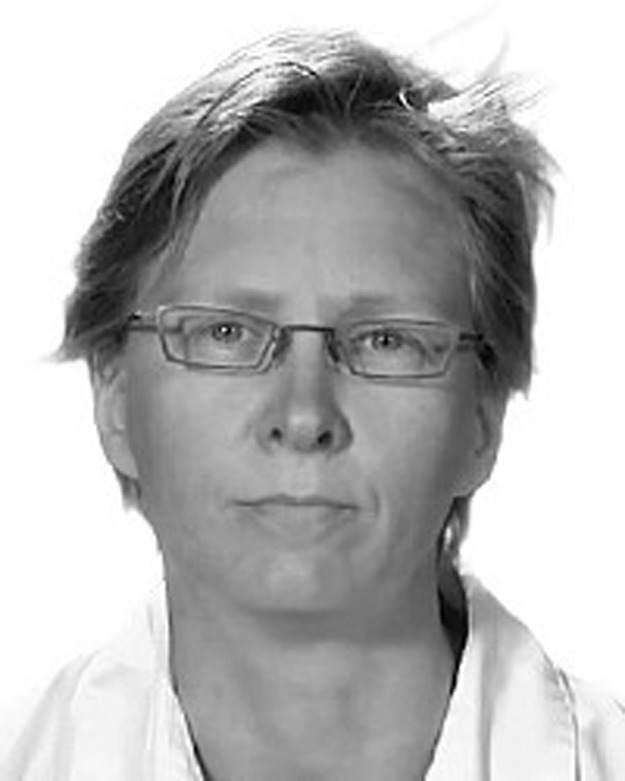
    Nicole Pouliart

**Nicole Pouliart** is head of the shoulder and elbow surgery unit, sports traumatology and arthroscopic surgery of the Department of Orthopaedics and Traumatology of the University Hospital of Brussels. She is also Associate Professor of Anatomy, Orthopaedics and Traumatology, and responsible for clinical reasoning and internships at the VUB Faculty of Medicine and Pharmacy. She graduated magna cum laude as a Medical Doctor at the Vrije Universiteit Brussel with in 1993, was orthopaedic trainee from 1993 to 1999 and obtained the Orthopaedic Board Certification in 1999. She was fellow in Intensive Care Medicine at the Academic Hospital of the Vrije Universiteit Brussel from 1999 to 2000 and obtained the Intensive Care Medicine Board Certification in 2002. She was also research fellow at Institut d’Anatomie de l’Université René Descartes à Paris V, France. She obtained a PhD in Medical sciences in 2005 on “Shoulder instability. Experimental Model and Related Anatomy”. She is involved in academic societies as SECEC (member of Research & Development Committee from 2006 to 2014 and chair since 2016), ABA (President), Belgian Elbow Shoulder Surgery BELSS, ISAKOS, ESSKA, BVOT. She is associate editor of the *Journal of Shoulder and Elbow Surgery* (JSES). She obtained different international awards from 1999 to 2007.

**Maryam Shahabpour** is Head of Clinics of Musculoskeletal Imaging and Sports Radiology at Universitair Ziekenhuis Brussel and Clinical Professor of Radiology at Vrije Universiteit Brussel (VUB) since 2007. She has been and is still Chair of the Sports Committee of the European Skeletal Society of Radiology (ESSR) since 2013. She is an active member of the International Skeletal Society (ISS) after being MSK Program Committee Chair in 2014–2015. She participates in the outreach programs of the ISS in Middle-East and Asia. She was MSK Scientific Committee member of the European Society of Radiology (ESR) in 2015–2016 and represents Belgium in the Education Committee of ESR since 2016.

She is organiser and coordinator of the Erasmus Courses on MRI (six times in Brussels from 1993 to 2016), and is board member since 2004 and secretary since 2008. She lectures since 1991 at all the MSK Erasmus courses (comprehensive course and course focused on joints). She was vice-president of the Annual Congress of ESSR in Bruges in June 2006 and organiser of the Radiological symposium of the EFOST Congress in Brussels in November 2010. She has been reviewer of *European Journal of Radiology* since 2004, *British Journal of Sports Medicine* and orthopaedic journals. Maryam Shahabpour has published 102 peer-reviewed papers (82 on PubMed), 73 chapters in radiological books and monographs (among them: co-editor and author of a few chapters in *Magnetic Resonance Imaging and Spectroscopy in Sports Medicine*, Springer-Verlag 1991 (translated in Japanese); MRI of the Knee in 2013 (University Publisher), MRI of the Ankle in 2016 (Breitenseher) and editor of three upcoming books (with Reto Sutter) on MRI of the shoulder, MRI of the elbow and MRI of the wrist, hand and fingers). She has presented 224 national and 243 international proffered and invited papers or refresher courses, including ESSR, ISS, ECR, RSNA and European sports and orthopaedic meetings.

The collaboration between the shoulder surgeon Nicole Pouliart and the MSK staff radiologists of UZ Brussel, Maryam Shahabpour, Michel de Maeseneer, Cedric Boulet and more recently Seema Doering started with a comparative study of CTA, MRA and arthroscopy in a large series of young patients with shoulder instability (supported by a research fund). Seeing the benefits of the regular radiosurgical confrontations, we came to the topic of today: “**What Can the Radiologist Do to Help the Surgeon Manage Shoulder Instability?**” Nicole and Maryam emphasize in a duo presentation why identification of abnormalities, whether variants or pathologic, is important to the surgeon facing a treatment decision and how important it is to collaborate with the clinician and to use the same language as the surgeon in our reports.

The typical lesions related with classic *anterior* and *anteroinferior* shoulder dislocation are an anteroinferior labral avulsion with or without bony fragment of bone loss (Bankart and Hill-Sachs lesions). These are relatively straightforward to identify on imaging, although normal variants of the inferior labrum and variants of labral damage may cause confusion. Other capsuloligamentous lesions, often associated with less typical types of instability, are much more difficult to correctly identify on imaging, as they occur in the *anterosuperior* part of the glenohumeral joint with its many normal variants or because they result in more subtle, and therefore easily overlooked changes in morphology or signal intensity. They try to answer to questions as: What does a normal labrum look like? Can we differentiate normal wear from pathology? Is the labrum always firmly attached to and flush with the glenoid rim? What are the signs of normal superior variants (11 to 1 o’clock)? or normal anterosuperior variants (1 to 3 o’clock)? How can we differentiate from pathology? Are there variants in the other areas: anterior, anteroinferior, and posterosuperior labrum? What shouldn’t we miss in patients with recurrent anterior dislocations, with capsulolabral and other lesions, with anterosuperior instability and rotator interval, MGHL and SGHL pathology, with biceps pulley lesions and biceps instability.

**2.** The **Chest Radiology** sessions are organized by **Walter De Wever** (Chair of the Chest Radiology Section of BSR) and the **Young Radiologist Section (YRS)**.

**2.1** The **first session on Chest imaging** is focused on **Pulmonary Embolism and Chest Pain** and moderated by Professor Walter De Wever (KUL- UZ Leuven).

He reports that pulmonary embolism is the third most common cause of cardiovascular death among Americans, behind myocardial infarction and stroke. The diagnosis or exclusion of pulmonary embolism as a cause of chest pain remains challenging for emergency physicians. Symptoms can be vague or non-existent, and the clinical presentation shares features with many other common diagnoses. Pulmonary embolism occurs when clots formed in the deep venous system dislodge or break loose, travel through the heart, and become lodged in the pulmonary vascular bed. The microvascular pulmonary bed has also the property to filter other tissue or substance like fat, air, tumor, septic material, organ fragments and foreign material. Also this can result in obstruction to blood flow.


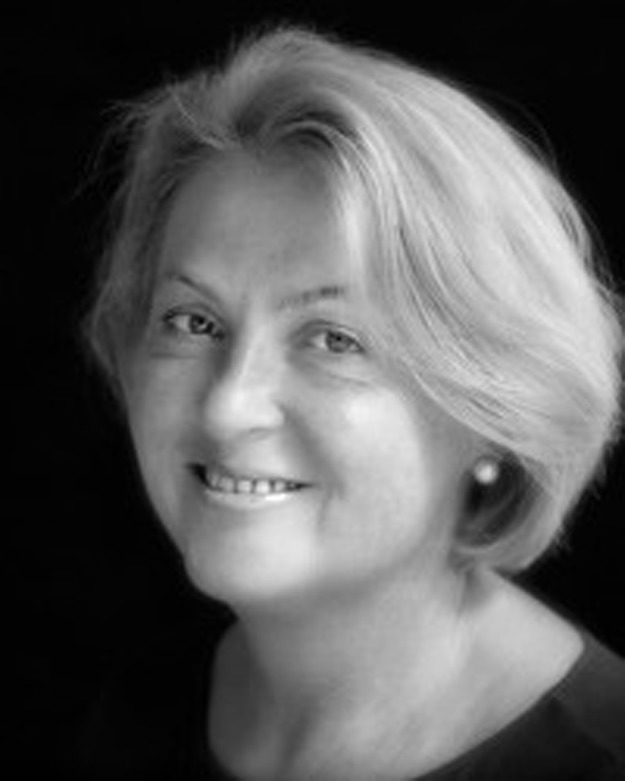
    Cornelia Schaefer-Prokop

The first topic on pulmonary embolism and chest pain is presented by Professor **Cornelia Schaefer-Prokop** (from Amersfoort, the Netherlands). She is radiologist at Meander Medical Centre, Amersfoort, and part-time researcher at DIAG. She has worked as radiologist in Hannover, Germany (1993–1998), AKH Vienna, Austria (1998–2004), AMC Amsterdam (2005–2009), and since 2009 in Meander Medical Centre Amersfoort. Her main research interests are in digital radiography, computer-aided detection, pulmonary embolism and interstitial lung diseases. She is editorial board member of *European Radiology, Journal of Thoracic Imaging* and *Insights into Imaging* and she is also a member of the Fleischner Society. She is the author of 110 peer reviewed publications (82 referred on PubMed) and editor of two books. She presents a lecture on “**Pulmonary Embolism, Subsegmental PE, Incidental PE: Diagnosis and Management**”.


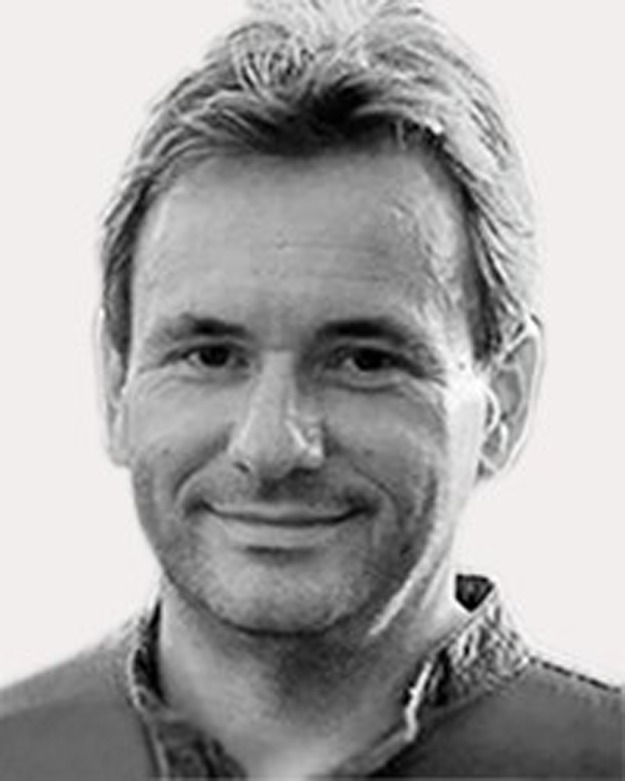
    Benoît Ghaye

The second speaker of this Chest Session is Professor **Benoît Ghaye**, Head of Clinics in the Radiology Department of the University Hospital Saint Luc (UCL) in Brussels since 2009. His main topic of interest is cardio-thoracic imaging. The topic of his PhD is “acute thrombo-embolic pulmonary disease”. He is a member of different scientific societies: ESTI, SIT, SRBR, SFR, RSNA, ESR and he is author of 28 peer-reviewed papers (referenced on PubMed and published in international journals as Radiology, Radiographics, AJR, European Radiology, Lancet, etc. He shares his experience on “**Non-thrombotic Pulmonary Emboli: Diagnosis and Management**” (see abstract).


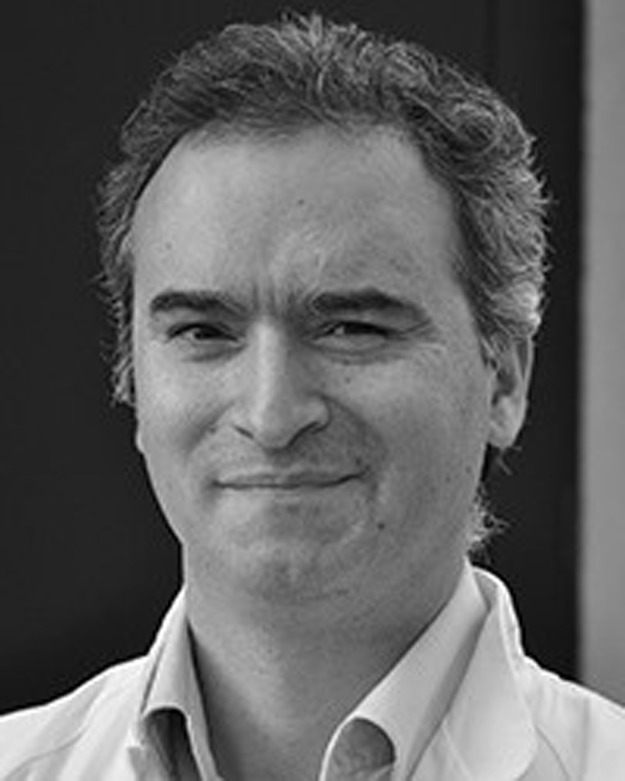
    Rodrigo Salgado

The third speaker is Professor **Rodrigo Salgado**, senior staff member at the Cardiovascular Imaging Unit of Antwerp University Hospital. He is former president of the Cardiac Imaging Section of the Belgian Society of Radiology and subcommittee board member of the European Society of Cardiac Radiology. He is reviewer for several international cardiovascular journals and member of the Scientific Council of the Belgian Society of Radiology. His current scientific research is focused on CT dose-reduction techniques, time-resolved MRA of vascular malformations and CT imaging of transcatheter heart valves. He has published more than 20 peer-reviewed papers (21 on PubMed) in international journals as Radiographics, AJR, JCAT, Cardiac and Thoracic Journals, etc.

He presents a lecture on “**Acute, Not PE-related Chest Pain: Diagnosis and Management**” (see abstract).

**2.2** The Young Radiologist Section has also prepared a session on **Chest Imaging: A Practical Approach**, chaired by Astrid Van Hoyweghen and Anne-Sophie Vanhoenacker (YRS members from respectively UZ Antwerp and KU Leuven).

**Anagha P. Parkar** (from Bergen, Norway) the first speaker of this session is invited by the YRS to present “**Differential Diagnosis of Cavitary Lung Lesions**”.


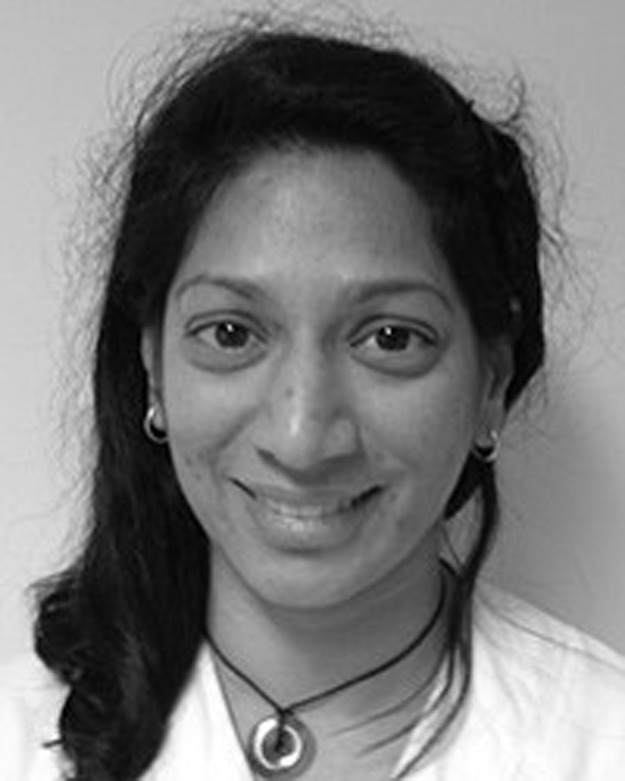
    Anagha Parkar

It is a topic that may be challenging in clinical settings, as there are many differential diagnoses (see paper). Since Anagha is working in the smaller hospital (of two) in Bergen, her presentation will be aimed at the level of trainees and general radiologists. She loves thoracic radiology, but as a “generalist”, she has to report on the whole body. Since 2008, she has been consultant radiologist at Haraldsplass Deaconess Hospital, Bergen, Norway. She graduated in 1999 at Ruhr Universität Bochum, Germany, and was certified Medical Doctor in Norway in 2001 and certified Radiologist in 2007. She has 8 peer-reviewed publications in international, Belgian and Norwegian journals, 12 invited lectures, 8 oral abstracts at international meetings and 13 posters. Since 2014 she has been secretary of the ESTI, team leader of the chest committee EDiR (written exam part) and chair of the Norwegian Society of Thoracic Radiology. In 2014, she obtained her ESSR diploma. She is Musculoskeletal Scientific Subcommittee member at ECR 2017 and 2018 and co-organiser of the Musculoskeletal Erasmus course on MRI of the Joints in April 2017 in Oslo, Norway. She organises in March 2016 the Spring meeting of the Norwegian Musculoskeletal Society and Spring meeting of the Norwegian Thoracic Radiology Society. She organised in April 2015 the Spring meeting of the Norwegian Thoracic Radiology Society and was local organiser of the Advanced MSK imaging ESMRMB in November 2013 and of the Norwegian Radiological Spring meeting in May 2006.


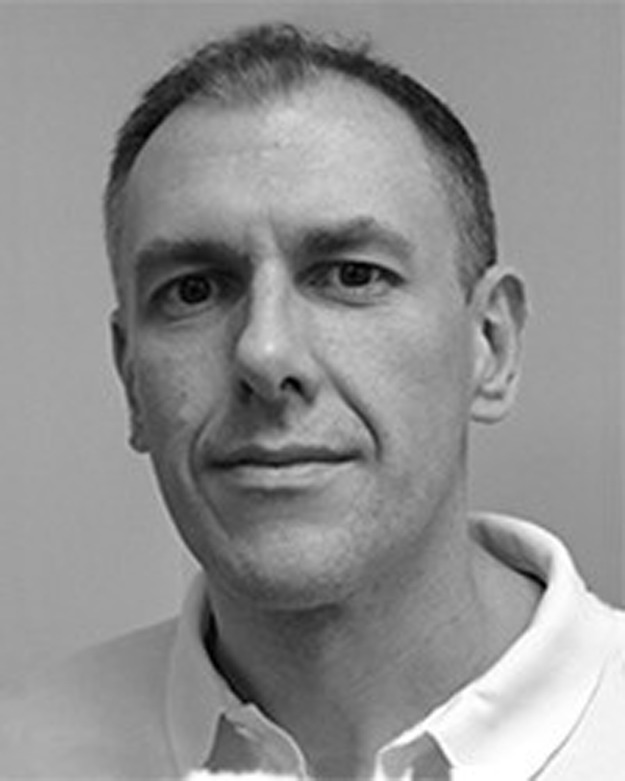
    Kris Van De Moortele

The second speaker of this session is Dr **Kris Van De Moortele** (from Bruges). He shares his expertise in “**Mediastinal Masses: Diagnostic Clues**”.


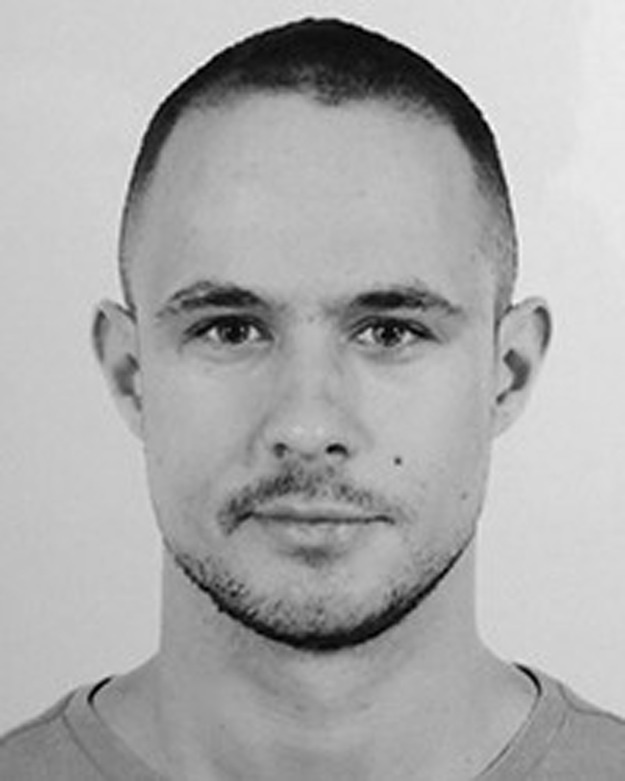
    Bart Ilsen

The third speaker of this chest session is **Bart Ilsen**. He graduated in Limburgs Universitair Centrum in 2003 and was resident in CHU-UVC Brugmann followed by AZ-VUB (Brussels) and was specialized in Radiodiagnosis in 2007. He has done 7 oral presentations in ESTI, ECR, WCTI, RSNA, ATS and published 12 peer reviewed articles in *Radiology, EJR, Emergency radiology*, etc., 7 being referenced in PubMed and 2 book chapters in *Comparative Interpretation of CT and Standard Radiography of the Chest*, edited by E Coche, B Ghaye, J de Mey and P Duyck (Springer-Verlag 2011).

He works as an expert one day a week for the ‘Fund for occupational diseases’, which entitles victims of occupational disease to a financial compensation. These diseases are often characterised by pleural pathologies and thus correspond well to the topic that he presents: “**Diseases of the Pleura and Chest Wall**”, with comparative interpretation of CT and standard radiography of the pleura. Many diseases affect the pleural space in both adults and children, including common diseases such as pneumonia, cancer and heart failure. Pleural effusion is the most common manifestation of pleural disease and it is often a secondary effect of another disease process. Doctor Ilsen emphasizes the crucial role of imaging in the management of pleural disease. Chest radiography remains often the first examination in the assessment of these patients. Depending on the clinical context, the optimal imaging technique for further evaluation might be computed tomography (CT), ultrasound (US) or magnetic resonance imaging (MRI).

The last speakers of this session are Dr **Laurent Van Camp** and Dr **Ward Vander Mijnsbrugge**, YRS members. They present an original case-based lecture on “**TB or Not TB: A Case-based Quiz**”.


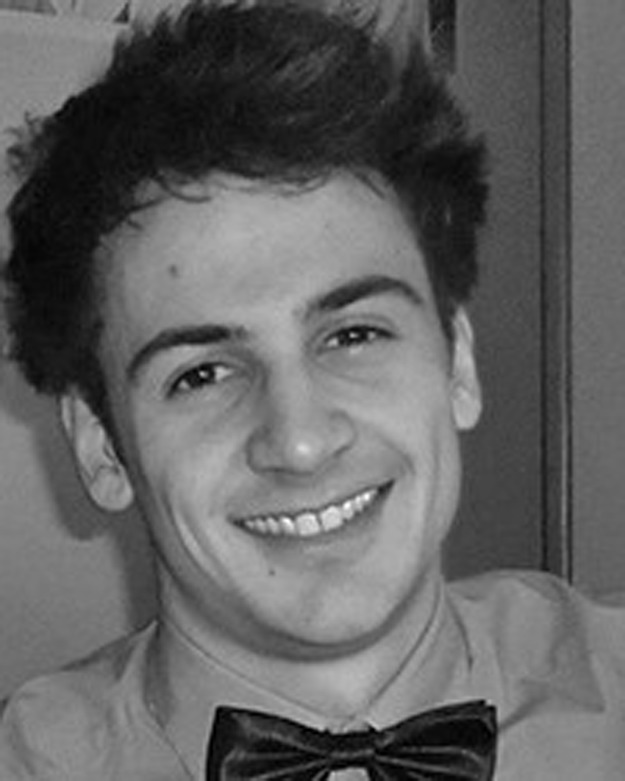
    Laurent Van Camp


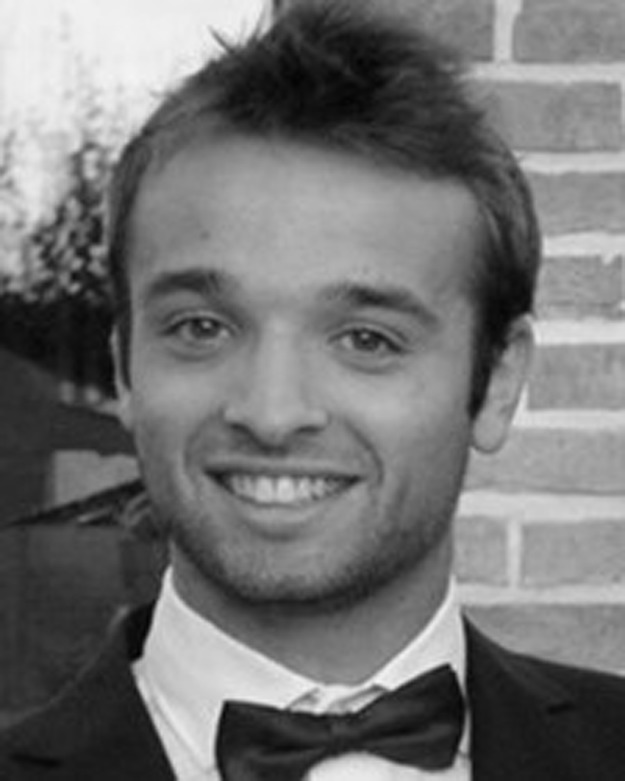
    Ward Vander Mijnsbrugge

**3. Plenary Session**

“**The BSR in 2016**” An overview of the most important activities is provided by BSR President Professor **Geert Villeirs**.


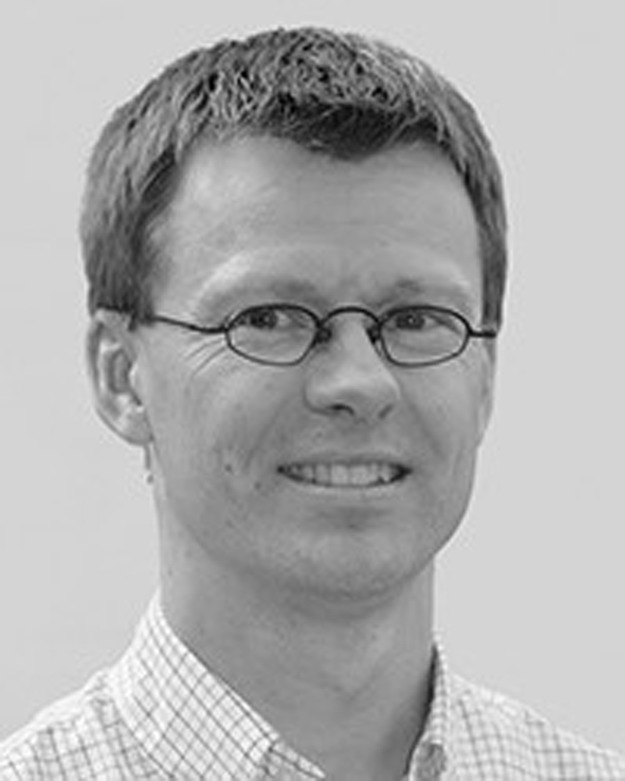
    Geert Villeirs

“**Keynote lecture**” by **Maggie De Block**, Federal Minister of Social Affairs and Public Health.

Maggie De Block will deliver a keynote address to all residents and radiologists at the BSR Annual Meeting 2016.

**4.** In the afternoon an **Image Interpretation Session so-called ‘Clash of the Titans’** is organized by the Young Radiologist Section with the help of the Thorax and MSK Belgian experts and is moderated by **Laurens Topff** and **Naïm Jerjir** (YRS/KUL).


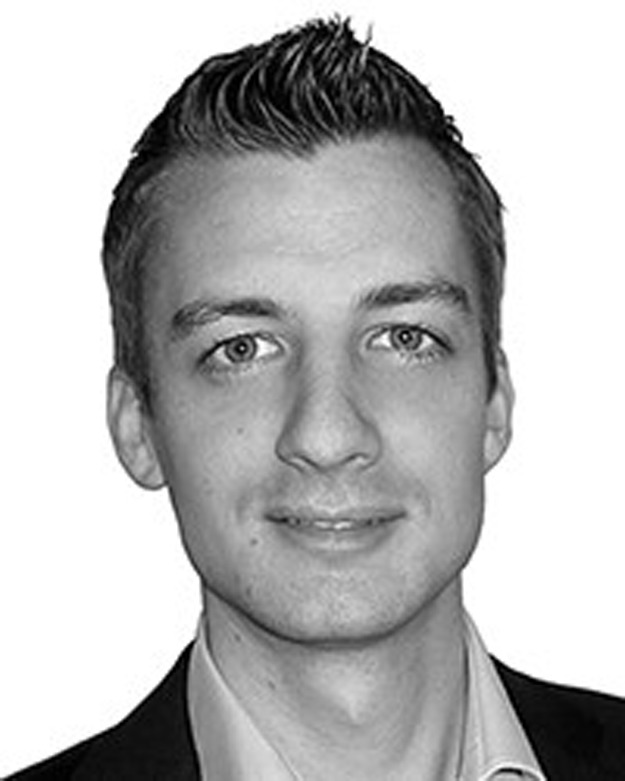
    Laurens Topff


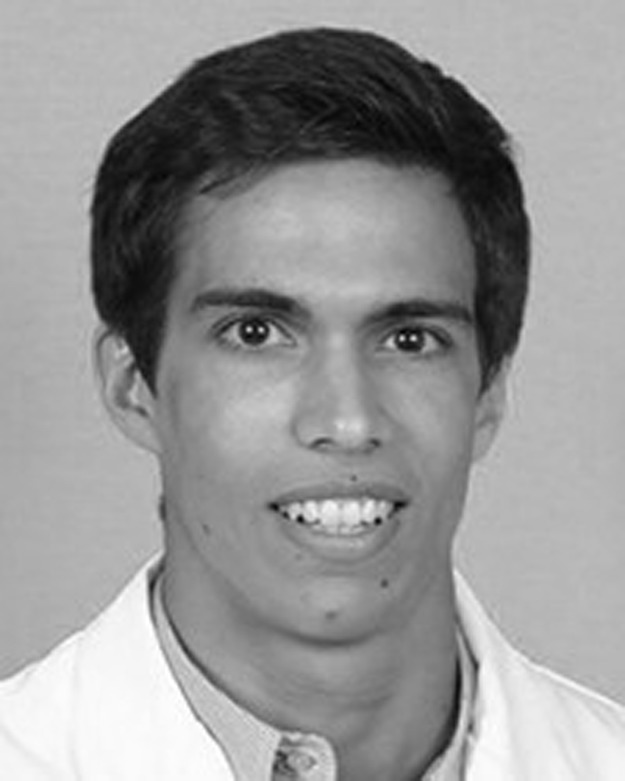
    Naïm Jerjir

The **MSK Expert panel** is represented by **Bruno Vande Berg** (UCL), **Filip Vanhoenacker** (UA-UG) and **Christian Glaser** (Munich,GE).

The **Chest Expert panel** is represented by **Johny Verschakelen** (KU Leuven), **Johan de Mey** (VUB)* and **Anagha Parkar** (Bergen, NO).

The purpose is to find out if the panel of experts can solve challenging cases and of course to learn from their interpretation skills.


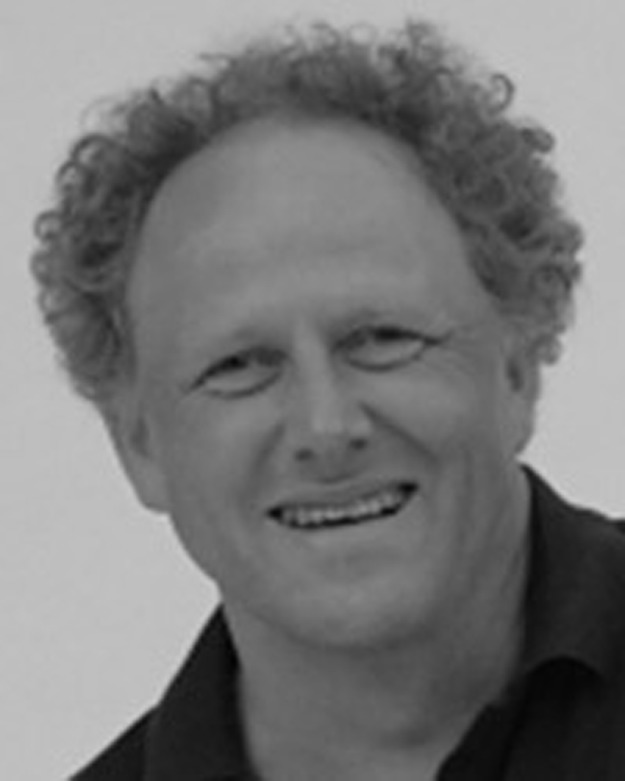
    Johan de Mey

*Professor **Johan de Mey** is Chair of the Department of Radiology in UZ Brussel and has been and is still Professor of Radiology at the VUB since 2005. He obtained a PhD on “CT-fluoroscopy in interventional radiology” and has 205 publications in peer-reviewed journals, over 300 presentations and 7 book chapters.

The BSR wishes you a very exciting meeting ☺

maryam.shahabpour@uzbrussel.be

